# Prenatal electroporation-mediated gene transfer restores Slc26a4 knock-out mouse hearing and vestibular function

**DOI:** 10.1038/s41598-019-54262-3

**Published:** 2019-11-29

**Authors:** Hiroki Takeda, Toru Miwa, Min Young Kim, Byung Yoon Choi, Yorihisa Orita, Ryosei Minoda

**Affiliations:** 1 Departments of Otolaryngology-Head and Neck Surgery Kumamoto University, Graduate School of Medicine, 1-1-1 Honjo, Chuoku Kumamoto city, Japan; 2 Department of Otolaryngology, University of Colorado Denver, Anschutz Medical Campus, Aurora, Colorado USA; 3 Departments of Otolaryngology-Head and Neck Surgery, Middle Ear and Inner Ear Surgical Center, JCHO Kumamoto General Hospital, 10-10 Tori Machi, Yatsushiro, Kumamoto 866-8660 Japan; 4 Department of Otorhinolaryngology, Seoul National University Bundang Hospital, Seongnam, South Korea

**Keywords:** Inner ear, Development

## Abstract

The otocyst, an anlage of the inner ear, presents an attractive target to study treatment strategies for genetic hearing loss and inner ear development. We have previously reported that electroporation-mediated transuterine gene transfer of *Connexin30*, utilizing a monophasic pulse into *Connexin30*^*−/−*^ mouse otocysts at embryonic day 11.5, is able to prevent putative hearing deterioration. However, it is not clear whether supplementary gene transfer can rescue significant morphological changes, caused by genetic deficits. In addition, with the transuterine gene transfer technique utilized in our previous report, the survival rate of embryos and their mothers after treatment was low, which became a serious obstacle for effective *in vivo* experiments. Here, we set out to elucidate the feasibility of supplementation therapy in *Slc26a4* deficient mice, utilizing biphasic pulses, optimized by modifying pulse conditions. Modification of the biphasic pulse conditions during electroporation increased the survival rate. In addition, supplementation of the target gene cDNA into the otocysts of homozygous *Slc24a4* knockout mice significantly prevented enlargement of the endolymphatic space in the inner ear areas; moreover, it rescued hearing and vestibular function of mice *in vivo*.

## Introduction

The prevalence of permanent childhood hearing loss, which is mainly due to a loss of cochlear function, is between 1.2 and 1.7 cases per 1,000 live births^1^, with genetic causes accounting for at least 50% of such cases in children^2^. Between 20 and 30% of affected children have profound hearing loss^3^, which has a life-changing impact, and the only current treatment modalities being hearing aids and cochlear implants. Hearing aids and cochlear implants are useful prostheses, but more fundamental therapeutic treatments for profound genetic hearing loss are required for example, stem cell-based or gene therapy.

Manipulation of the developing inner ear is an attractive experimental strategy for investigating treatment modalities for inner ear diseases and for studying the course of normal inner ear development. The otocyst, which is an anlage of the inner ear, may be the best target for such studies as it is a closed and isolated epithelial vesicle and is the origin of the anatomical structure of the adult inner ear^4,5^. There have been a limited number of reports describing direct manipulation of the otocyst in mammals *in vivo*, likely because otocyst inoculation in mammals requires intricate surgery; the otocyst cannot be directly visualized and it is necessary to manipulate the embryo through the uterine wall^6,7^. Previous reports have demonstrated successful otocystic inoculation of exogenous genes in mice using viral vectors^5^, or exogenous protein in mice using cell-penetrating peptides (CPPs)^8^. However, strategies utilizing viral vectors had several limitations, including low transfection efficiency, restrictions based on cell type, cellular toxicity, and/or requirement for optimization for each tissue. In addition, CPPs have the drawback of short transfection periods^8,9^. Meanwhile, there have been a few positive reports where electroporation is applied to the developing inner ear, utilizing monophasic non-attenuating multiple pulses^10,11^. Gubbels *et al*. reported that functional cochlear hair cells could be generated with electroporation-mediated *Atho1* gene transfection into the otocysts in mice *in vivo*^10^. In addition, Miwa *et al*. also reported that electroporation-mediated transfer of wild type gap junction *Connexin30* (*Cx30*) genes, which encode CONNEXIN (CX) 30 proteins, into the otocysts of *Cx30* deleted mice at E 11.5, were able to prevent putative hearing deterioration^11^. Thus, electroporation-mediated transuterine gene transfer into otocysts (EUGO) appears to be one of the most promising transfection methods for gene induction in the developing inner ear. However, the low survival rate of treated embryos presents a drawback to electroporation-based transfection, and this is also a limiting factor for achieving effective *in vivo* experiments. Additionally, it has yet to be determined whether this treatment concept is applicable to genetic hearing loss, which is caused by different mechanisms and displays significant morphological changes in the inner ear. Furthermore, the absence of functional CX30 did not appear to cause major morphological changes initially^12^ and thus rescue of major morphological changes has never been tested by this method. Therefore, the first goal of the present study was to increase the survival rate of treated embryos exhibiting high gene/protein expression. The second goal of this study was to clarify whether supplementary gene transfer into otocysts can rescue more significant morphological changes caused by genetic deficits.

Regarding the first goal, it has been recently reported that increasing pulse amplitude during electroporation increases transfection rate and decreases survival rates of treated embryos after intraventricular plasmid injection^13^, or plasmid injection into fertilized eggs^14^. Electroporation utilizing multiple attenuating biphasic pulses, which consist of poring pulses (Pp) and transfer pulses (Tp), may be another option for achieving more successful gene transfer^15–19^. Yamono *et al*. reported that higher Pp and longer pulse length increased DNA transfection *in vitro*^15^. They also found that the reducing amplitudes of second Pp and Tp by 40% can increase the transfer rate up to 26 fold when compared to monophasic pulses^15^. Thus, modification of conditions for electroporation-mediated gene transfer may improve survival and transfection rates in treated otocysts.

Genetic hearing losses are generally divided into two subgroups, non-syndromic and syndromic. The most common form of syndromic sensorineural hearing loss is Pendred syndrome, which is associated with developmental abnormalities of the inner ear, and diffuse thyroid enlargement^20^. Its most prominent morphological feature in the inner ear is vestibular aqueduct enlargement^21^. Therefore, we set out to clarify the second goal of the present study utilizing a mouse model of this condition. Approximately half of Pendred syndrome cases are associated with mutations within the solute carrier family 26 (*SLC26A4*) gene^20,22,23^. *SLC26A4* encodes PENDRIN protein, which is an anion exchanger that is expressed in non-sensoriepithelial cells in the cochlea, vestibule and endolymphatic sac (ES)^24^. In mice, PENDRIN is firstly expressed in the ES at E 11.5, in the cochlear hook-region at E 13.5, in the utricle and the saccule at E 14.5, in the basal turn of the cochlea and the ampulla at E 16.5, and in the upper turn of the cochlea at E 17.5^25^. *Slc26a4* deleted mice display an enlargement of the endolymphatic space followed by a failure to develop normal hearing and balance^25,26^.

Choi *et al*. have previously demonstrated temporal regulation of *Slc26a4* expression in transgenic mouse lines employing tetracycline-inducible system (Tet-On). They revealed that expression of *Slc26a4* is necessary throughout a developmental period E 16.5 to P 2 for acquisition of normal hearing. In addition, the preventive efficacy against hearing loss diminishes when the gene is expressed after E 16.5^24^. Thus, temporal expression of *Slc26a4* during E 16.5 to P 2 may be sufficient for rescuing the phenotype caused by *Slc26a4* deletion. This suggests that the genetic manipulation of the developing inner ear of *Slc26a4* deleted mice can be a treatment strategy. We set out to determine optimal electroporation conditions by modifying parameters of monophasic and biphasic pulses first, and then to elucidate feasibility of the supplementation therapy in *Slc26a4* deficient mice, utilizing optimized pulses in EUGO.

## Results

### Modifying parameters of electroporation pulses

**Figure 1:**
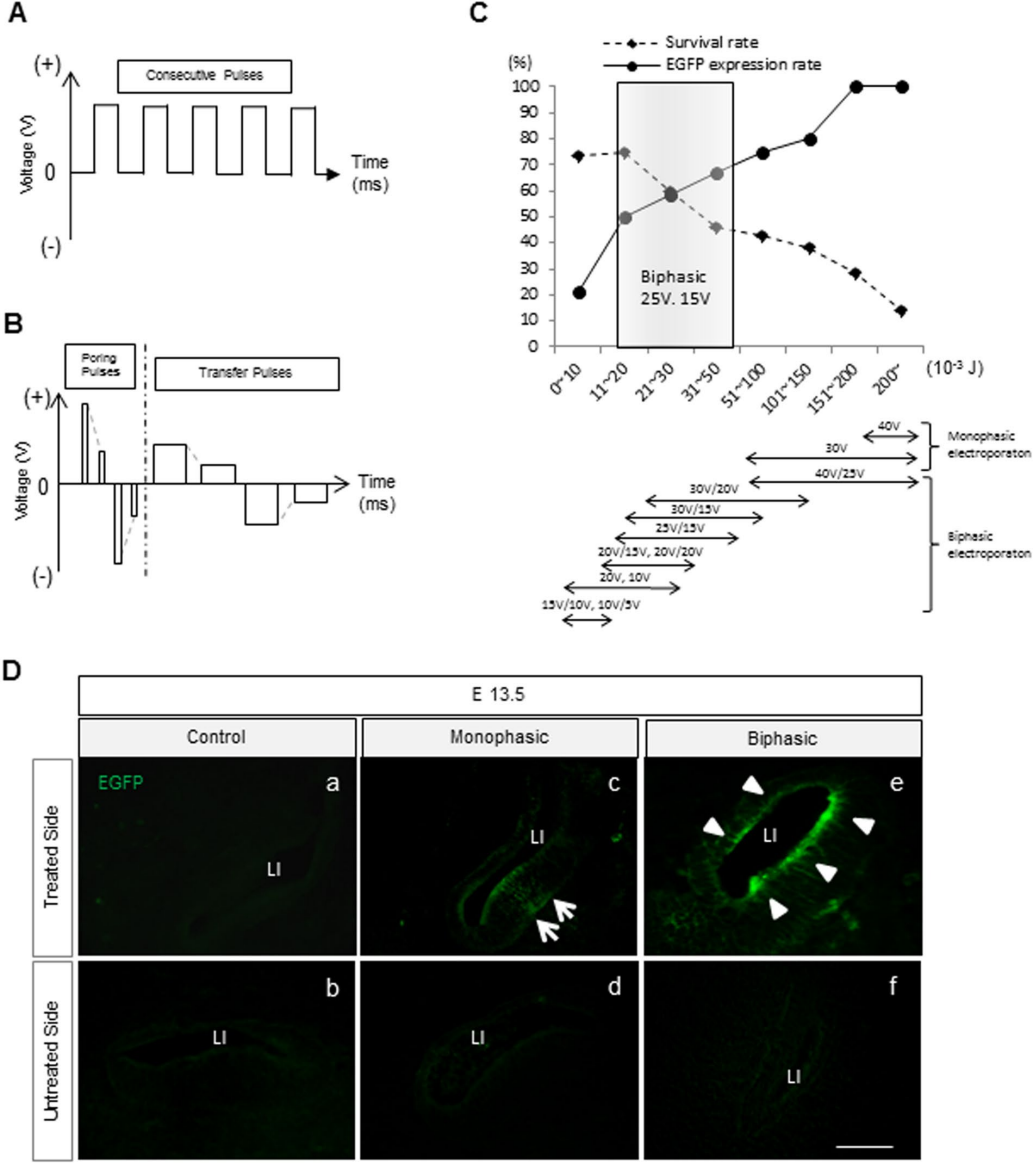
Gene transfection methods via electroporation, in WT mice. (**A**) Schematic of the electroporation conditions of monophasic (M-) pulses. (**B**) Schematic of the electroporation conditions of biphasic (B-) pulses. (**C**) The graph shows survival and EGFP expression (number of animals who had EGFP expression in treated animals) rate of E 13.5 treated mice in M and B-treated mice. X-axis represents total energy (10^*−*3^ J) in different electroporation settings. (**D**) Sectional images at E 13.5 of control (a,b), M-treated group (c and d), and B-treated group (e and f). The arrows show unilateral EGFP expression of the treated cochlear epithelium in the M-treated group (c), and the arrowheads indicate EGFP expression of the whole treated cochlear epithelium in the B-treated group (e). Scale bar indicates 50 μm.

To determine the optimized pulse condition regarding both survival and expression rates, EGFP expression was assessed at E 13.5 by fluorescent microscope after treatment with monophasic (M) (Fig. 1A) and biphasic (B) pulses (Fig. 1B) at E 11.5, respectively (Supplemental Table 1). In the M-treated group, overall survival rate in all conditions was 35.7% (Supplemental Table 2B). Survival rate improved when the total energy was reduced, but this resulted in a decrease of the EGFP expression (Fig. 1C and Supplemental Table 2B). In the B-treated group, overall survival rate was 57.1%, and the proportion of EGFP expression of the treated inner ear epithelium was 46.7% (Supplemental Table 2C). Among the various conditions of Pp and Tp, the best condition for highest survival and EGFP expression rates in treated embryos was Pp 25 V and Tp 15 V and over 50% of this condition showed 21–31 × 10^*−*3^ J where survival rate and EGFP expression rate are crossing (Fig. 1C). Electroporation utilizing this condition showed an 77.8% of survival rate and 55.6% of positive EGFP expression in treated embryos (Supplemental Table 2C).

In the control (plasmid filled but non-electroporated) group, survival rate of treated dams was 83.3%, and EGFP expression rate was 0% as expected (Supplemental Table 2A).

### EGFP protein expression and auditory function after electroporation

In the control group, we did not observe any EGFP signal in the inner ear at E 13.5 (Fig. 1Da,b). In the B-treated group, EGFP was detectable in the lining cells and in the vicinity of the lining cells of the treated inner ear at E 13.5 (Fig. 1De,f), while in the M-treated group EGFP was detectable on mainly one side of the lining cells in the treated inner ear (Fig. 1Dc,d). Auditory function was assessed after EUGO utilizing biphasic pulses on WT mice at P 30. There was no significant difference in the auditory thresholds at 4,000, 12,000, or 20,000 Hz between the treated and non-treated side (Supplemental Fig. 1), indicating that the EUGO procedure has no significant harmful effect on hearing for the treated mice.

### Effects of gene transfection on *Slc26a4*^*−*/*−*^ mice

**Figure 2:**
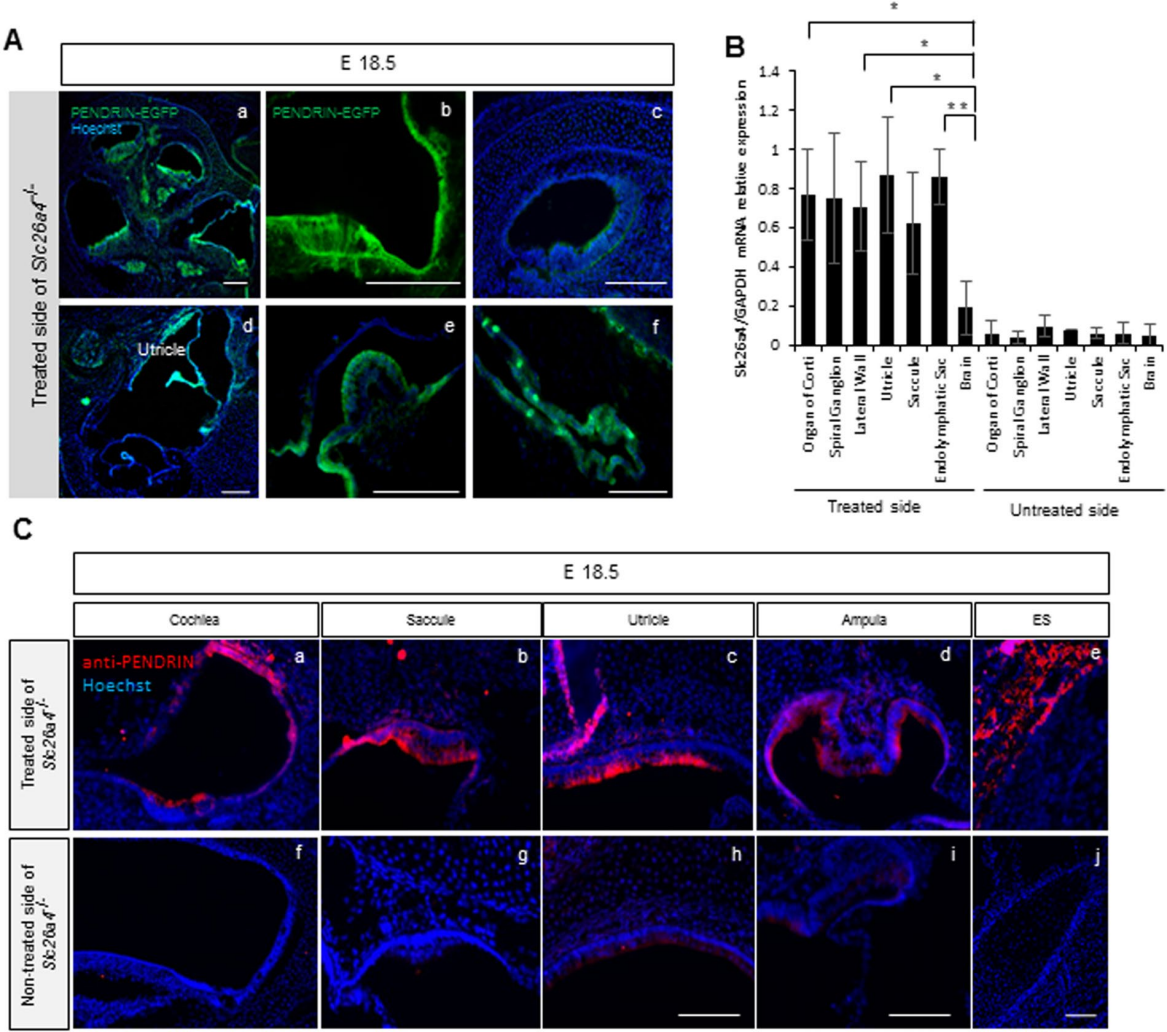
The results of histology and qPCR after treatment at E 18.5. (**A**) Images of the embryonic inner ear at E 18.5 in the treated side of *Slc26a4*^*−*/*−*^ mice. PENDRIN-EGFP expression is detectable at the lateral wall in the cochlear middle turn (a,b), the utricle (d), the ampulla of semicircular canals (e) and the ES (f), while hardly in the cochlear apical turn (c). Green indicates PENDRIN-EGFP expression, and blue indicates nuclear (Hoechst) staining. Bars represent 100 μm. (**B**) *Slc26a4* mRNA expression in each region of the inner ear by quantitative rtPCR from LMD. The bars represent mean *Slc26a4* mRNA expression levels against *GAPDH* mRNA expression levels. *n* = 5. ^*^P < 0.05. ^**^P < 0.01 (Mann-Whitney U). (**C**) Immunohistochemistry of the embryonic inner ear at E 18.5 in the treated (a–e) and non-treated (f–j) sides of *Slc26a4*^*−*/*−*^ mice utilizing anti-PENDRIN antibody. PENDRIN expression are seen widely in the cochlea (a), saccule (b), utricle (c), ampulla (d) and endolymphatic sac (ES) (e) in the treated side of *Slc26a4*^*−*/*−*^ mice. There was no PENDRIN signal seen in the non-treated side of *Slc26a4*^*−*/*−*^ mice. Red indicates PENDRIN expression and blue indicates nuclear (Hoechst) staining. Bars represent 50 μm.

For gene transfection in *Slc26a4*^*−*/*−*^ mice otocysts at E 11.5, we utilized 25 V of Pp and 15 V of Tp as this pulse condition showed the highest survival and EGFP expression rates as described above. Overall survival rate at E 18.5 and P 30 of the treated PENDRIN KO mice was 50% and 46.9% respectively. At E 18.5, after treatment at E 11.5, naïve PENDRIN-EGFP signal and PENDRIN expression using anti-PENDRIN was detectable at the lateral wall and the organ of Corti in cochlear middle and basal turn (Fig. 2A,C), the utricle (Fig. 2Ad,C), the saccule (Fig. 2C), the ampulla of semicircular canals (Fig. 2Ae,C), and the endolymphatic sac (ES) (Fig. 2Af,C) by histology; this expression pattern was observed from almost all of the regions or more regions where endogenous PENDRIN protein is originally expressed (Supplementary Fig. 2). However, EGFP was not clearly detected at the cochlear apical turn at E 18.5 (Fig. 2Ac).

Furthermore, we confirmed *Slc26a4* gene expression at E 18.5 utilizing quantitative real time PCR. Each tissue (the organ of Corti; OC, Spiral Ganglion; SG, lateral wall; LW, utricular macula; U, saccular macula; S, endolymphatic sac; ES and brain; Br) was collected using laser microdissection (LMD) at E 18.5 after treatment in *Slc26a4*^*−*/*−*^ mice (Supplemental Fig. 3). Relative expression of *Slc26a4* mRNA in each sample revealed that the gene was expressed in the treated side of OC, SG, LW, U, S, ES, and Br, while those genes was not clearly detectable in the non-treated side of U, S, ES and Br. There were no significant differences in relative mRNA expression level in among OC, SG, LW, U, S and ES in the treated inner ear, while there were significant differences between Br and OC, LW, U or ES; which suggests that the transfected gene level was almost equal among OC, SG, LW, U, S and ES in the treated side (Fig. 2B).

**Figure 3:**
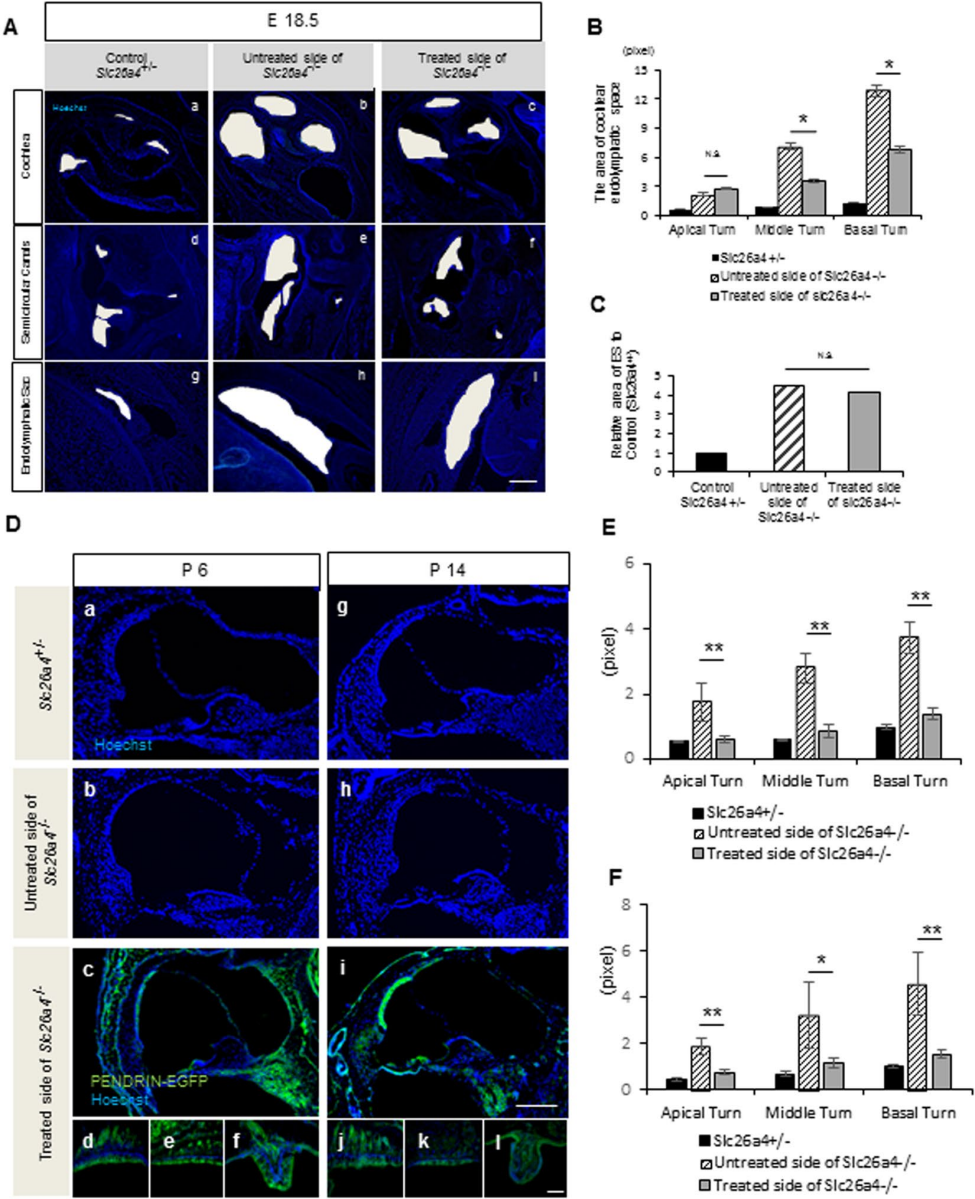
The results of immunohistochemistry and areas of endolymphatic space in the cochlear turns at E 18.5, P 6 and P 14. (**A**) Images after treatment at E 18.5 in the treated side of *Slc26a4*^*−*/*−*^ (c,f,i), non-treated side of *Slc26a4*^*−*/*−*^ (b,e,h), and *Slc26a4*^+/*−*^ control mice (a,d,g). White regions indicate the space of the endolymphatic space of the cochlea (a–c), semicircular canals (d–f), and endolymphatic sac (g–i). Blue; Hoechst. (**B**) The graph shows the size of the area in the scala media (SM) at each cochlear turn at E 18.5. The SM at basal and middle turn in the treated side is significantly smaller than that in the non-treated side. *n* = 5. *P < 0.05 (Mann-Whitney U). (**C**) The graph showing the relative size of the area in the endolymphatic sac (ES) to that in the non-treated control. There is no significant difference between the treated side and the non-treated side. *n* = 5. *P < 0.05 (Mann-Whitney U). Scale bars indicate 100 μm. (**D**) Images showing PENDRIN-EGFP is detectable at the stria vascularis, organ of Corti and Rosenthal’s canal in the cochlea (c,i), saccular macula (d,j), utricular macula (e,k), the ampulla of semicircular canals (f,l) in the treated *Slc26a4*^*−*/*−*^ inner ear at both P 6 and P 14. The areas of endolymphatic space in the cochlear middle turns of the treated side of *Slc26a4*^*−*/*−*^ cochlea at both P 6 and P 14 are smaller than that of the untreated side of *Slc26a4*^*−*/*−*^ cochlea. Green indicates PENDRIN-EGFP, and blue indicates Hoechst. Scale bars indicate 100 μm. (**E**,**F**) The graphs show the areas of endolymphatic space in the cochlear turns at P 6 and P 14 respectively. The areas are significantly reduced in all cochlear turns in the treated mice when compared with that in the untreated side. *n* = 5. *P < 0.05. **P < 0.01 (Mann-Whitney U).

The degree of ballooning in the cochlear scala media (SM) was assessed at E 18.5 (Fig. 3A,B). We excluded the vestibular systems from the statistical analysis for this assessment as the number of samples was low, although the degree of ballooning in the treated side appeared to be slightly lower than that the non-treated *Slc26a4*^*−*/*−*^ semicircular canals (Fig. 3Ad–f). The SM area in the basal and middle turn in the treated ear was significantly smaller than that of the non-treated side, while SM area in the apical turn and ES in the treated ear showed no significant difference compared to those in the non-treated side (Fig. 3B,C).

**Figure 4:**
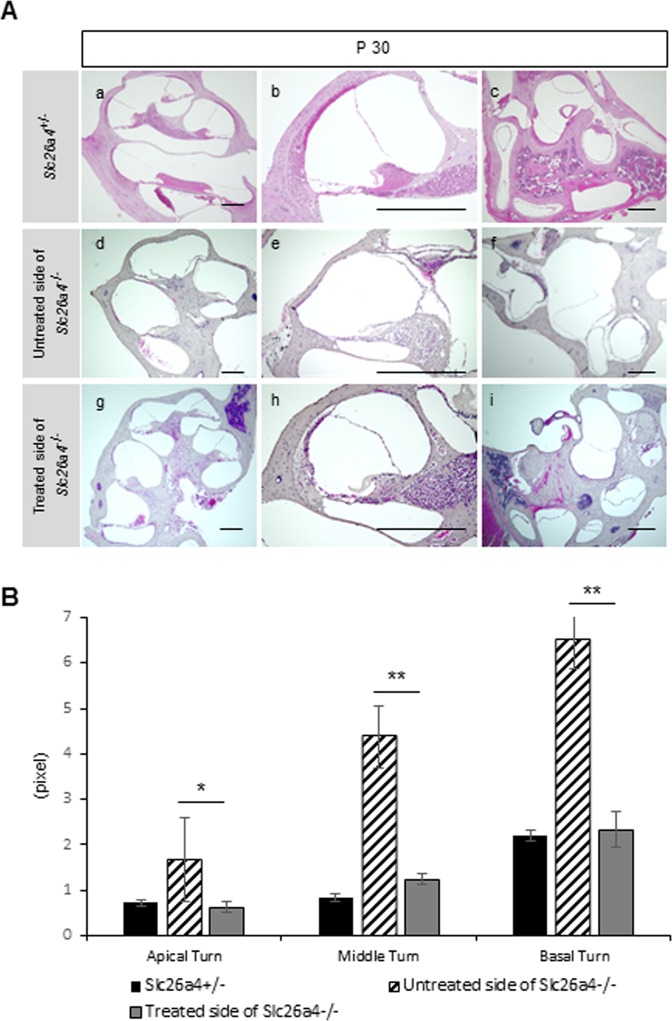
Morphology (H&E staining) and areas of endolymphatic space in each cochlear turn at P 30. (**A**) Sectional images of the *Slc26a4*^+/*−*^ mice (a–c), the untreated side of *Slc26a4*^*−*/*−*^ mice (d-f) and the treated side of *Slc26a4*^*−*/*−*^ mice (g–i) at P 30. The areas of endolymphatic space of the treated side of *Slc26a4*^*−*/*−*^ are smaller than that of the untreated side of *Slc26a4*^*−*/*−*^. Scale bars represent 100 μm. (**B**) The graph shows the areas of endolymphatic space in each cochlear turn at P 30. Each area of three turns in the treated cochleae is significantly reduced when compared with that in the untreated side. n = 5. ^*^P < 0.05. ^**^P < 0.01 (Mann-Whitney U).

We also evaluated EGFP expression in treated inner ears of postnatal mice histologically and post-natal ballooning of the endolymphatic space morphologically. At P 6 and P 14, EGFP was still detectable in the cochlea (especially in the OC, stria vascularis and spiral ganglion), saccule, utricle and ampulla of the semicircular canals (Fig. 3D). The degree of ballooning of SM in the treated *Slc26a4*^*−*/*−*^ cochlea at P 6, P 14 and P 30 was lower than that in untreated cochlea in cross section (Figs. 3D and 4A). Moreover, the SM area in all cochlear turns in the treated ears was also statistically smaller than that of the non-treated side at each time point (Figs. 3E,F and 4B). At P 30, the degree of ballooning in the vestibular system of the treated side in *Slc26a4*^*−*/*−*^ mice also appeared to be lower than that in the untreated side in sliced sections, although statistical analysis was not performed (Fig. 4A).

**Figure 5:**
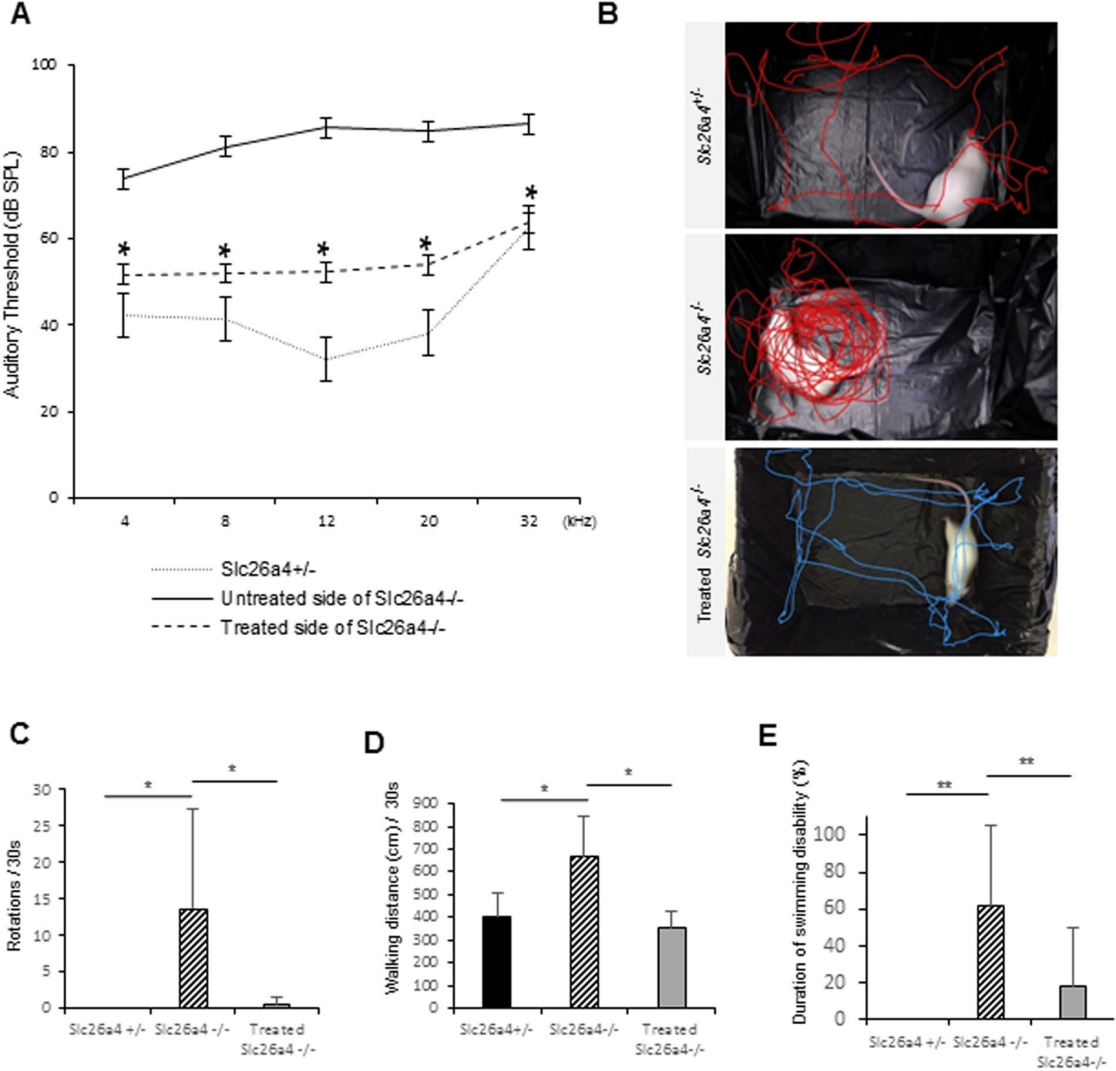
The results of auditory function tests and vestibular function tests at P 30. (**A**) ABR testing results. Statistically significant difference is found between the treated and the untreated sides in *Slc26a4*^*−*/*−*^ mice at 4, 8, 12, 20 and 32 kHz. Each *n* = 5. ^*^P < 0.05 (Mann-Whitney U). (**B**) Representative track plots for 15 seconds. The *Slc26a4*^*−*/*−*^ mouse is severely circling, while *Slc26a4*^+/*−*^ and treated *Slc26a4*^*−*/*−*^ mice are not circling and walking almost normally. (**C**) This graph shows numbers of rotation per 30 seconds. *n* = 5. ^*^P < 0.05 (Mann-Whitney U). (**D**) This graph shows walking distance per 30 seconds. *n* = 5. ^*^P < 0.05 (Mann-Whitney U). (**E**) This graph shows duration of swimming disability. Statistically significant difference is found between the non-treated *Slc26a4*^*−*/*−*^ mice and the treated *Slc26a4*^*−/−*^ mice as for numbers of rotation, walking distance and duration of swimming disability. *n* = 5. ^*^P < 0.05. ^**^P < 0.01 (Man*n*-Whitney U).

In order to test function after treatment in *Slc26a4*^*−*/*−*^ mice, vestibular function tests were performed at P 30, and ABR was conducted at P30 and P90. The ABR threshold in treated ears was significantly improved from that in non-treated ears across all frequencies, 4, 8, 12, 20 and 32 kHz (Fig. 5A) and the difference between treated and non-treated ears continued until P 90 at 4, 8 and 12 kHz (Supplemental Fig. 5). Rotational frequency and walking distance in treated *slc26a4*^*−*/*−*^ mice were significantly decreased compared to that in non-treated *slc26a4*^*−*/*−*^ mice at P 30 (Fig. 5B–D and Supplemental Fig. 4). Moreover, the duration of swimming disability was also significantly reduced in treated *slc26a4*^*−*/*−*^ mice (Fig. 5E). These results indicate that hearing and vestibular function of treated *slc26a4*^*−*/*−*^ mice at P 30 were significantly better than non-treated *slc26a4*^*−*/*−*^ mice despite that a full rescue was not achieved.

## Discussion

We have previously shown that EUGO utilizing monophasic non-attenuating pulses is a useful experimental procedure to study treatment strategies for genetic hearing loss^11,27^, although a notable drawback of this procedure was low survival rate of the treated embryos. To resolve this issue, we utilized multiple attenuating biphasic pulses for electroporation instead of monophasic pulses, which were previously reported^10,11^. Consequently, the modification of amplitudes of Pp and Tp in multiple attenuating biphasic pulses showed positive effects on the survival rates in treated embryos. We observed a maximum survival rate of 77.8% in the treated embryos when utilizing a condition of Pp 25 V x2 and Tp 15 V x2; this rate was nearly equal to the survival rate in the control group. This demonstrated that causes of remaining survival failure rate of treated embryos were probably damages by otocystic plasmid injection, surgery and/or pinching with paddles during surgeries. To reduce this rate, we need to develop less invasive manipulation technique for surgeries, or alternatively less invasive gene transfer methods, while maintaining high transfection rate for possible future applications in human patients. Meanwhile in our study, the higher amplitude appears to cause higher damage in the embryos. Considering our results, Pp 25 V × 2 and Tp 15 V × 2 is considered the optimal condition for our EUGO system.

When considering the treatment of pathologies caused by autosomal recessive, loss-of-function variants, an ideal optimal treatment strategy should faithfully simulate initiation timing, location and duration of normal gene expression in the inner ear in order to compensate for the loss of function^27^. Choi *et al*. have reported that forced expression of *Slc26a4* in transgenic mice during E 16.5 to P 2, using Tet-On system, completely prevents subsequent hearing loss^24^. Their data suggest that induced, pulse expression of *Slc26a4* throughout the critical window of development (namely, E 16.5 to P 2) with the same spatial expression pattern as that of endogenous *Slc26a4*, may be sufficient for preventing subsequent hearing loss and disequilibrium^24^. However, such precise control of temporal and spatial expression patterns of target genes is technically difficult to replicate in gene therapy *in vivo*. We have previously reported successful prevention of auditory system deterioration that was achieved through EUGO of *Cx30* cDNA in *Cx30*-deleted mice at E11.5^11^; normal *Cx30* initial expression begins around E 14^28^. The expression area of exogenous *Cx30*, which was obtained via EUGO of normal *Cx30* cDNA, was wider than the area of endogenous *Cx30* gene expression in wild type mice. These data implies that precise matching of temporal and spatial expression patterns of supplemental gene to those of normal gene may not be always necessary to achieve positive therapeutic effects in *Cx30* deleted mice. In the present study, we similarly performed EUGO of normal *Slc26a4* cDNA to homozygous *Slc26a4* deleted mice utilizing biphasic pulses at E 11.5, a same time point as normal gene initiation in wild type mice. We induced PENDRIN expression at wider areas than those observed in non-treated *Slc26a4*^+/*−*^ cochleae. Consequently, the supplementation of *Slc26a4* cDNA into the otocysts of *Slc26a4* deleted mice significantly attenuated enlargement of the endolymphatic space in the inner ear areas and prevented post-natal hearing loss and vestibular dysfunction in treated mice. Our present results are consistent with previously reported data^11,24^. However, collateral effect of this study which resulted in ectopically expression of PENDRIN protein in the cells where shouldn’t normally have PENDRIN protein should be considered though we did not find any ototoxicity.

In our study, we observed significant decreases in the endolymphatic areas in the middle and basal turns at E 18.5 when compared with untreated cochlear data, while there was no significant area difference in the endolymphatic space in the cochlear apical turns between treated and untreated cochlea. This finding might be a reflection of the characteristics of the embryonic cochlea development. During mouse gestation, the cochlea starts to elongate at E 10.75, and the elongation is completed by E 17^29^. Therefore, it happened that in the present study, most of the cochlear elongation process took place after otocystic gene transfer, and there was only 1.5 day between the completion of the elongation process of the cochlea and E 18.5. Further, it is known that the apical turns are formed later than the basal turns. Therefore, it is conceivable that the gene expression level of *Slc26a4* of the basal turn would have more benefited from this gene transfer than that of the apical turn at E 18.5. In turn, this speculative lower gene expression level of *Slc26a4* at the apical turn at E 18.5 may have led to our results. To interest, the endolymphatic space enlargement in the apical turns was rescued at later stages such as P 6, P 14 and P 30. We sought to clarify the reason for the discrepancy in the apical turns and at later stages. It could be that the cochlear apical turn at earlier time points might not have sufficient time to respond to gene transfer. Therefore, *Slc26a4* transgene expression might not have had any significant effect against the endolymphatic space enlargement at the tip of elongating cochlea, however, it could have obtained significant effects at more advanced stages along the development of the cochlea. Indeed, auditory function at 8, 12, 20, and 32 kHz (which reflects apical-basal cochlear turn) in the treated side and vestibular function of *Slc26a4* deficient mice were both rescued by EUGO as evaluated at P 30. Alternatively, the *Slc26a4* transgene expression may have differential intrinsic effects on attenuation of the endolymphatic space enlargement dependent on the developmental stage of the cochlea.

In this study, ES enlargement was not rescued after otocystic gene transfer by EUGO, while EGFP, PENDRIN protein and *Slc26a4* mRNA expression was detectable in the ES at E 18.5. Since we did not evaluate the ES status at postnatal stages, we could not exclude the possibility that the ES may be downsized at such later stages, as was the later restoration of the cochlear apical turn. This issue warrants further study. Conversely, the PENDRIN expression level in the ES at E 18.5 in the present study may be insufficient to rescue the enlargement of ES, although we have not definitively established the sufficient *slc26a4* expression level required to rescue enlargement of the ES. Meanwhile, Li *et al*. previously reported that transgenic mice that have *Slc26a4* expression only in the ES showed no enlargement of the membranous labyrinth and normal hearing and vestibular function^30^. This result suggests that *Slc26a4* expression in the ES is key to rescuing morphological and functional abnormalities caused by *Slc26a4* deficiency. Given the hearing and vestibular functional rescue from our study, rescue of the enlargement of the ES was strongly expected, however, our results at least at E 18.5 did not coincide with this expectation. Considering these results, we speculate that *Slc26a4* expression in the ES, but not the rescue of the enlargement of the ES would be necessary for functional rescue of vestibular and hearing deficit. In accordance with our speculation, it has been previously reported that patients with enlargement of the vestibular aqueducts do not always have hearing loss and vestibular dysfunction^31,32^.

In summary, EUGO utilizing biphasic pulses appears to be a more efficient transfection strategy of target genes when comparing EUGO utilizing monophasic pulses. Supplementary treatment via EUGO utilizing biphasic pulses to *Slc26a4* deficient mice significantly reduced ballooning changes in SM at all turns of the cochlea. Moreover, in treated mice, hearing and vestibular function were partially rescued. The present results show that, without precise matching to the gene expression areas, we can obtain amelioration of functional deficits by gene therapy via EUGO in mice with genetic inner ear dysfunction if expression of transfected genes covers the critical window period of gene function. When considering future clinical applications of the present treatment concept, we propose further refinement of the gene transfection methods in order to increase treatment efficacy and safety.

## Methods

### Animals and genotyping

Timed-pregnant wild type (WT) CD-1 mice were purchased from Japan SLC Inc. (Shizuoka, Japan), and original 129 Sv/Ev strain *Slc26a4* knockout (*Slc26a4*^*−*/*−*^) mice were generated by NIH^26^ (by Dr. Eric Green). The *Slc26a4*^*−*/*−*^ on 129 Sv/Ev background mice was backcrossed with WT mice on a CD-1 background until 3^rd^ generation. 3^rd^ generation *Slc26a4*^+/*−*^ mice were maintained by crossbreeding *Slc26a4*^+/*−*^ mice × *Slc26a4*^+/*−*^ mice or *Slc26a4*^+/*−*^ mice × *Slc26a4*^*−*/*−*^ mice. Noon on the day on which a vaginal plug was detected was designated as E 0.5 of development. E 11.5 timed-pregnant *Slc26a4*^+/*−*^ mice, which were crossbred with male *Slc26a4*^*−*/*−*^ mice or male *Slc26a4*^+/*−*^ mice were used for EUGO experiments. Treated pups were genotyped at the same point as morphological assessments were performed, utilizing the Extract-N-Amp™ Tissue PCR Kit (Sigma, St. Louis, MO). Primers for genotyping of *Slc26a4* mutants were: TGCCGATTTCATCGCTGG, GCATTGTAGTTCTTTTCCAAGTTGG and GGGTGCGGAGAAAGAGGTAATG.

### Recombinant plasmids

*Egfp* plasmid vectors were purchased from GeneCopoeia (EGFP control vector p.Reciever-M02, Rockville, MD). The plasmids express EGFP proteins under the CMV promoter with an SV 40 PolyA signal. We generated *Slc26a4*-*Egfp* plasmid vectors by inserting mouse *Slc26a4* cDNA into “BamH and Sal1” sites to the p.EGFP-N1 (Catalog#6085-1; Clontech, Mountain View, CA). The concentration of both *Egfp* and *Slc26a4-Egfp* vectors were adjusted to 1.0 µg/µl.

### Plasmid injection procedure

For plasmid inoculation into the mouse otocyst, E 11.5 timed-pregnant mice were anesthetized with intraperitoneal administration of 10 mg/kg of xylazine (Bayer, KS) and 80 mg/kg of ketamine–HCl (Daiichisankyo, Tokyo, Japan) in saline solution. Procedure details were described previously^11^. Briefly, the uteri were gently removed from the pregnant mice following low midline laparotomy, *Egfp*-plasmid or *Slc26a4*-*Egfp* plasmid vectors with 0.1% fast green (Sigma, Missouri) as a tracking dye were injected by oral pressure into one side of otocysts in one or two embryos per a dam using heat-pulled glass micropipettes whose tip diameters were from 10 to 15 µm. Subsequently, the plasmid-filled otocysts were pinched on both sides of the embryonic head using a tweezer-style electrode paddle, then electroporated.

### Electroporation

In order to determine optimized pulse conditions, plasmid-filled otocysts were electroporated in WT mice utilizing two methods: monophasic multiple pulses (M-treated group) (Fig. 1A) and multiple biphasic pulses, which consist of Pp and Tp (B-treated group) (Fig. 1B). In M-treated group, the electroporation was achieved utilizing CUY21 EDIT (NEPAGENE, Chiba, Japan). The parameters were as follows: voltages were 30 or 40 V: pulse length was 50 ms: pulse interval was 950 ms: and number of pulses was 5 (Fig. 1A and Supplemental Table 1A). In B-treated group, the electroporation was achieved utilizing NEPA21 (NEPAGENE, Chiba, Japan). The multiple biphasic pulses consist of biphasic poring pulses (Pp) with attenuated amplitudes, which makes a hole in the cell membrane, and biphasic transfer pulses (Tp) with attenuated amplitudes, which allows the plasmid to move into the cytoplasm. We changed Pp voltages from 10 to 40 V, and Tp voltages from 5 to 25 V. The other parameters of Pp were as follows: pulse length was 10 ms; pulse interval was 950 ms; number of pulses was 2; and decay rate from each antecedent pulse was 40%. The other parameters of Tp were as follows: pulse length was 50 ms; pulse interval was 950 ms; number of pulses was 2; and, decay rate was 40% (Fig. 1B and Supplemental Table 1B). In the control group, plasmid-filled otocysts were not electroporated after otocystic plasmid injections. After electroporation, the embryos were returned to the abdominal cavity and the abdominal skin of treated dams was sutured.

### Experimental protocol

For determining the optimal pulse conditions, treated embryos were histologically assessed at E 13.5 from both M- and B-treated groups. Survivability of the treated embryos was evaluated after anesthetizing their mothers, and EGFP expression of surviving embryos was assessed by visual inspection of EGFP fluorescence in the inner ear by immunohistochemistry. For postnatal evaluation, the embryos, which were delivered via c-section at E 18.5, were extracted and surrogated to other mothers. To determine the feasibility of supplementary treatment for *Slc26a4*^*−/−*^ mice, treated *Slc26a4*^*−*/*−*^ embryos which were injected *Slc26a4-Egfp* plasmids and electroporated at E 11.5 using biphasic electroporation were harvested at E 18.5, P 6, P 14 and P 30 for further analysis.

### Cryosectioning

For assessments of immunohistochemistry at E 13.5, E 18.5 and P 6, extracted inner ears were fixed in 4% paraformaldehyde (PFA) overnight at 4 °C. For postnatal assessments at P 14 and P 30, the inner ears were decalcified for 2–4 days at room temperature in EDTA solution (Wako, Osaka, Japan) after fixation. Subsequently, the tissues were embedded in OCT medium and were serially cryosectioned at 8 μm. For hematoxylin and eosin (H&E) staining at P 30, the fixed and decalcified tissues were transferred to 70% ethanol and embedded in paraffin medium, and serially cryosectioned to a thickness of 5 μm.

### Immunohistochemistry and area evaluation

Cryosectioned slices were counterstained with Hoechst 33258 dye (Molecular Probes, OR, USA) for 30 sec for nuclear staining after fixing with 4% PFA for 15 minutes and blocking with 0.1% Triton-X (IBI Scientific, Peosta, IA) in phosphate buffer saline (PBS) for 20 min. During each process, the tissues were washed three times for 5 min each with PBS. We evaluated naive EGFP expression without any antibody and utilized anti-PENDRIN primary antibody (1:50, sc-23779 Santa Cruz) and Alexa 555 secondary antibody (1:500, Thermo Fisher) for detecting PENDRIN protein expression. For H&E staining at P 30, histological staining was performed using H&E to assess gross morphology. All samples were observed and captured under a BZ-9000 fluorescence microscope (Keyence, Osaka, Japan). All images were captured using the same photographic exposure conditions at a resolution of 1360 × 1024 pixels. In the *Slc26a4*-related experiment, we randomly selected two sliced sections per cochlea from serially sliced sections which included the modiolus and one apical turn, two 2^nd^ turns and one basal turn images. On the captured images, we manually selected outlines of the scala media (SM) at each cochlear turn and areas of the endolymphatic sac (ES) and calculated the size (pixels) utilizing Image J software (NIH, MD). We divided the ES area for treated cochleae (at each turn) by the area for non-treated cochleae. This analysis was performed in a blinded manner (*n* = 5 in the treated cochleae, and *n* = 10 in the non-treated *Slc26a4*^+/*−*^ cochleae).

### Laser microdissection (LMD)

At E 18.5 after treatment of *Slc26a4*^*−/−*^ mice, whole heads were dissected and incubated in 4% PFA for 1 h at room temperature. They were embedded in OCT compound, then sectioned at 10 μm thickness in the plane of the long axis of the cochlear modiolus. Sections were mounted on uncharged slides (Leica, Wetzlar, Germany) and dried at room temperature. The slides were incubated in acetone at −20 °C, then dried at room temperature immediately prior to performing LMD. LMD was performed using the LMD7 system (Leica). Samples containing cells were obtained from the organ of Corti, Rosenthal’s canal, cochlear lateral wall, utricular macula (U), saccular macula (S), and endolymphatic sac (ES). Each slide contained multiple adjacent sections, and we pooled all cells in each category from individual slides onto a single cap. As controls, brain (Br) of the treated side, and U, S, ES and Br of the non-treated side (Uc, Sc, ESc, Brc respectively) were obtained. Sampled brain tissues were collected from the cerebral cortex close to the sampled cochleae.

### Quantitative real time-PCR (RT-PCR)

Total RNA was extracted from each sample excised with LMD using a micro RNA Extraction kit (Qiagen, Valencia, CA). After the isolation steps, we quantified RNA using a GeneQuant100 (GE Healthcare Ltd., Amersham, UK) and equalized each sample. The cDNA was synthesized from total RNA with SuperScript^®^ VILOTM Master Mix (Life technology, Carlsbad, CA), following manufacturers instruction. For quantitative RT-PCR, primers for *Slc26a4* or *GAPDH* (Takara Bio., Otsu, Japan) and templates were mixed with SYBR^®^ Green PCR Master Mix (Life technology). The cDNA was amplified for 40 cycles of denaturation for 15 sec at 95 °C and annealing for 1 min at 60 °C, using a Takara thermal cycler Dice system (model TP960). The relative gene expression of *Slc26a4* and of *GAPDH* was calculated by the standard curve method. Transcript levels of *Slc26a4* were normalized to *GAPDH*.

### Auditory brainstem response (ABR)

We assessed the impact of EUGO utilizing biphasic pulses on hearing of WT mice at P 30 and P 90 after *Egfp*-plasmids treatment in B-treated group, and assessed the efficacy of gene supplemental therapy on *Slc26a4*^*−*/*−*^ mouse auditory function after treatment. Auditory thresholds were determined using auditory brain stem responses (ABR) (ABR System 3: Tucker-Davis Technologies, Alachua, FL). Briefly, the animals were anesthetized by the intraperitoneal administration of mixture of xylazine and ketamine. Electrodes were placed beneath the pinna of the treated ear and at the vertex just below the surface of the skin, and then the ground electrode placed under the contralateral ear. An average of 1,024 sweeps was calculated for 4,000, 12,000 and 20,000 Hz in WT mice, and an average of 512 sweeps was calculated for 4,000, 8,000, 12,000, 20,000 and 32000 Hz for *Slc26a4*^*−/−*^ mice. The stimulus levels near the threshold were varied in 5-dB steps, and the threshold was defined as the lowest level at which waves in the ABR could be clearly detected by visual inspection.

### Vestibular function test

At P 30 in treated *Slc26a4*^*−/−*^, untreated *Slc26a4*^*−/−*^ and control *Slc26a4*^*+/−*^ mice, we performed two types of vestibular function test: circling behavior test and the swimming test. To test circling behavior, the animals were placed in 20 × 16 cm black boxes and their movement in the box was captured in mp4 type video (1080p at 30fps), then analyzed using the freeware motion-analysis software Kinovea (version 0.8.15, available for download at: http://www.kinovea.org), which tracked the center of the animal’s eyes as they moved. The total distance (cm) was divided by time (30 seconds): distance/30 sec. In the same way, frequencies of rotation per total time was measured. To test swimming ability, the animals were placed in warm water-filled boxes for 5 to 31 seconds, then we measured the percentage of swimming disability (we defined the **“**swimming disability” as drowning and/or circling in water). We compromised on measuring long time for untreated *Slc26a4*^*−/−*^ mice because of their severe drowning in order to avoid undesired adverse effects. The total time (sec) of swimming disability was divided by total swimming time (sec) to obtain percentage of duration of swimming disability (%).

### Statistics

All of the data were statistically analyzed utilizing the Mann-Whitney U test between two independent groups. Data are presented in the text and in figures as mean ± SD. The statistical significance level was set at a p value less than 0.05.

### Study approval

All animal experiments were approved by the Committee on the Use and Care of Animals at Kumamoto University and were performed using accepted veterinary standards.

## Supplementary information

Supplementary file
