# Supplementary material for: Prenatal electroporation-mediated gene transfer restores Slc26a4 knock-out mouse hearing and vestibular function
Source: Sci Rep. 2019 Nov 29;9:17979. doi: 10.1038/s41598-019-54262-3 (PMC6884448; doi:10.1038/s41598-019-54262-3)
Supplement: Supplementary file 1 — Supplementary file [file 41598_2019_54262_MOESM1_ESM.pdf]

Prenatal electroporation-mediated gene transfer restores Slc26a4 knock-out mouse hearing and vestibular function

Supplemental figures, tables and legends

Hiroki Takeda[1,2], Toru Miwa[1,3], Min young Kim[4], Byung Yoon Choi[4], Yorihisa Orita[1] and Ryosei Minoda*[3]

[1]: Departments of Otolaryngology-Head and Neck Surgery Kumamoto University, Graduate School of Medicine, 1-1-1 Honjo, Chuoku, Kumamoto city, Japan

[2]: Department of Otolaryngology, University of Colorado Denver, Anschutz Medical Campus, Aurora, Colorado, USA

[3]: Departments of Otolaryngology-Head and Neck Surgery, Middle Ear and Inner Ear Surgical Center, JCHO Kumamoto General Hospital, 10-10 Tori Machi, Yatsushiro, Kumamoto, 866-8660, Japan

[4]: Department of Otorhinolaryngology, Seoul National University Bundang Hospital, Seongnam, South Korea

A Electroporation utilizing a Monophasic Pulse

Voltage (V)	Pulse Width (ms)	Pulse Interval (ms)	Frequency
30 or 40	50	950	5

B Electroporation utilizing Biphasic Pulses

Poring Pulses（Pp）						Transfer Pulses（Tp）					
**Voltage** (V)	Pulse Width (ms)	Pulse Duration (ms)	Frequency	Attenuation (%)	Polarity	**Voltage** (V)	Pulse Width (ms)	Pulse Duration (ms)	Frequency	Attenuation (%)	Polarity
10 to 40	10	950	2	40	+/-	5 to 25	50	950	2	40%	+/-

Supplemental Table 1

Pulse conditions in the M-treated group **(A)** and in the B-treated group **(B)**.

A Control

Voltage (V)	Survival Rates of Treated Dams	Survival Rates of Treated Embryos	EGFP Expression Rates in Survived Embryos	EGFP Expression Rates in Treated Embryos
0	83.3%(5/6)	83.3% (5/6)	0.0% (0/0)	0.0% (0/0)

B Electroporation utilizing a Monophasic Pulse

Voltage (V)	Survival Rates of Treated Dams	Survival rates of treated embryos	EGFP expression rates in survived embryos	EGFP expression rates in treated embryos
40 x5	100% (4/4)	12.5% (1/8)	100.0% (1/1)	12.5% (1/8)
30 x5	100% (3/3)	66.7% (4/6)	75.0% (3/4)	50.0% (3/6)
Total	100% (7/7)	35.7% (5/14)	80.0% (4/5)	21.4% (3/14)

C Electroporation utilizing Biphasic Pulses

Pp Voltage (V)	Tp Voltage (V)	Survival Rates of Treated Dams	Survival Rates of Treated Embryos	EGFP expression rates in survived embryos	EGFP expression rates in treated embryos
40 x2	25 x2	62.5% (5/8)	10.0% (1/10)	0.0% (0/1)	0.0% (0/10)
30 x2	20 x2	100% (3/3)	50.0% (3/6)	33.0% (1/3)	16.7% (1/6)
**25 x2**	**15 x2**	100% (5/5)	**77.8%** (7/9)	**71.4%** (5/7)	**55.6%** (5/9)
20 x2	20 x2	100% (3/3)	50.0% (2/4)	50.0% (1/2)	25.0% (1/4)
20 x2	15 x2	100% (5/5)	77.8% (7/9)	42.9%(3/7)	33.3% (3/9)
20 x2	10 x2	100% (3/3)	66.7% (4/6)	50.0% (2/4)	33.3% (2/6)
15 x2	10 x2	100% (4/4)	62.5% (5/8)	25.0% (1/4)	12.5% (1/8)
10 x2	5 x2	100% (3/3)	75.0% (3/4)	0.0% (0/3)	0.0% (0/4)
Total		91.2% (31/34)	53.6% (30/56)	46.7% (14/30)	25.0%(14/56)

Supplemental Table 2.

The survival rates and EGFP expression rates after treatment in the control group **(A)**, in the M-treated group **(B)**, and in the B-treated group **(C)**.

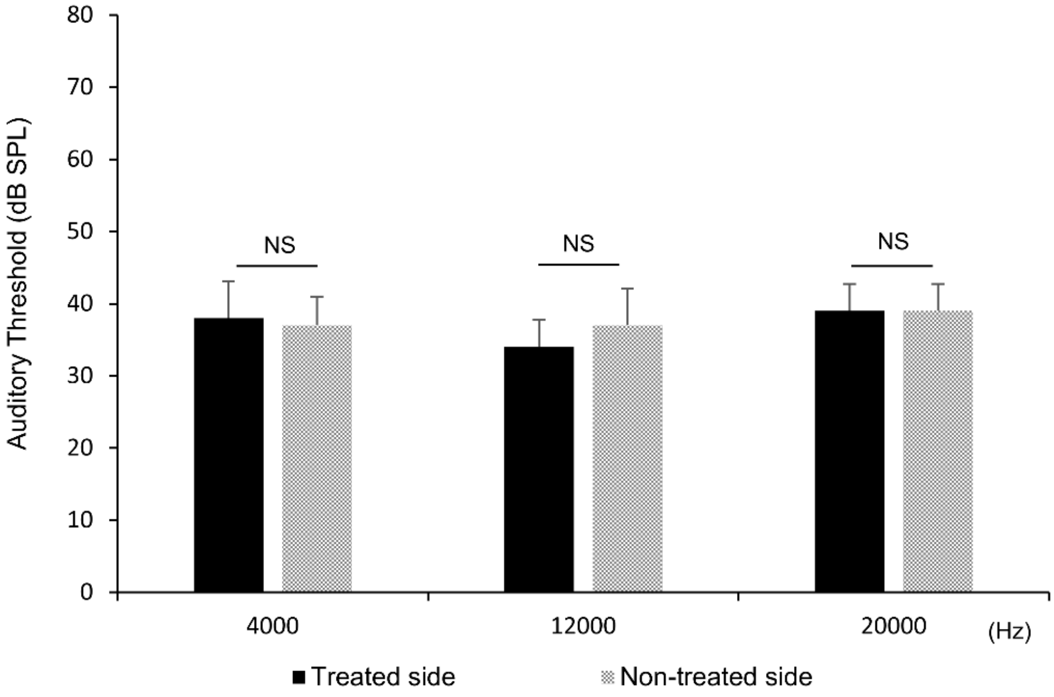

Supplemental Figure 1.

ABR testing results of WT mice at P 30 after *Egfp* gene transfection in the B-treated group. No significant difference is found between the treated and the non-treated sides at 4, 12 and 20 kHz. *n*=5.

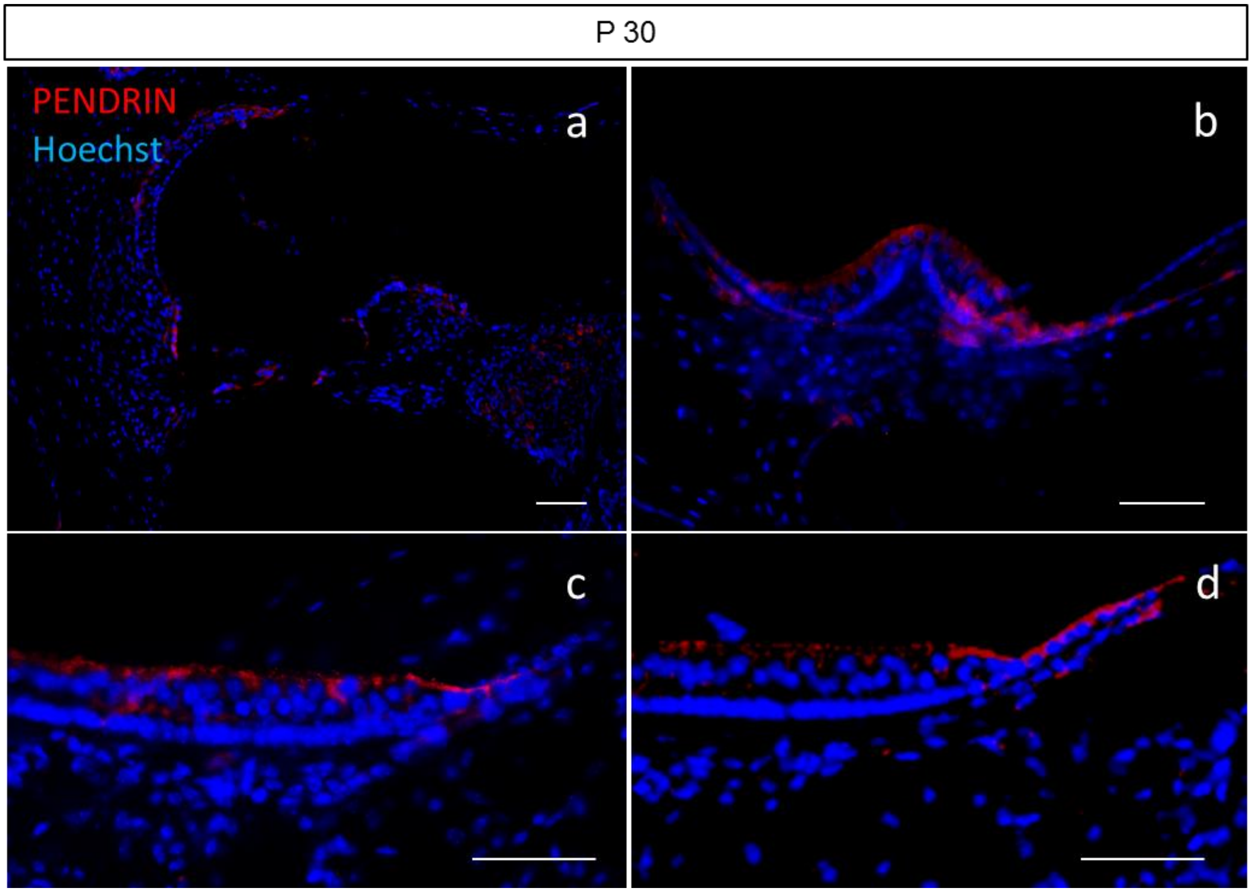

Supplemental Figure 2.

Immunohistochemistry of the wild type mouse inner ear at P 30 using anti-PENDRIN antibody. PENDRIN expressions are found in the cochlea (a), ampulla (b), saccule (b) and utricle (c). Red indicates PENDRIN expression and blue indicates nuclear (Hoechst) staining. Bars represent 50 μm each.

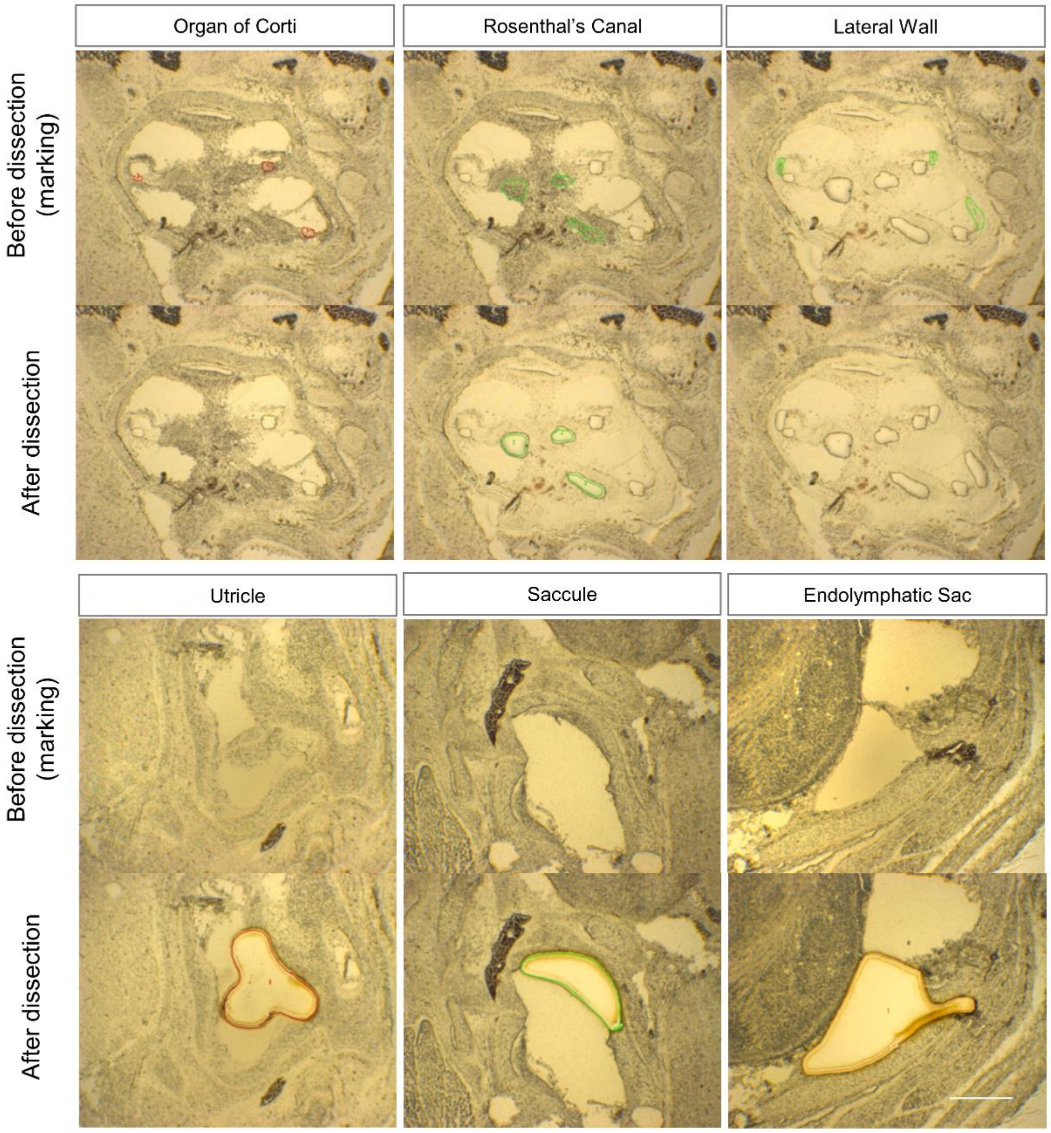

Supplemental Figure 3.

Nomarski images for LMD. The areas surrounded by yellow-green or orange colored lines show respective areas. Bar indicates 100 μm.

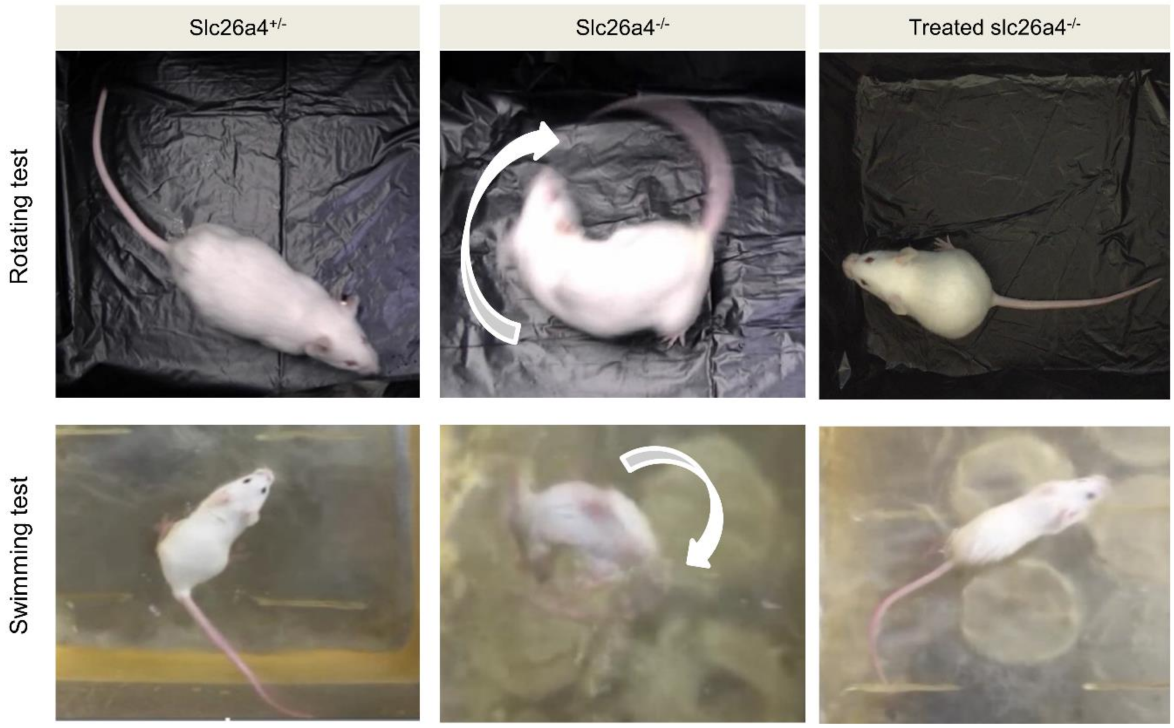

Supplemental Figure 4.

Images of the *Slc26a4*$^{+/-}$, non-treated *Slc26a4*$^{-/-}$ and treated *Slc26a4*$^{-/-}$ mice in the box or water at P 30. Non-treated *Slc26a4*$^{-/-}$ mice display severe circling behavior and swimming disability, while *Slc26a4*$^{+/-}$ and treated *Slc26a4*$^{-/-}$ mice behave and swim almost normally.

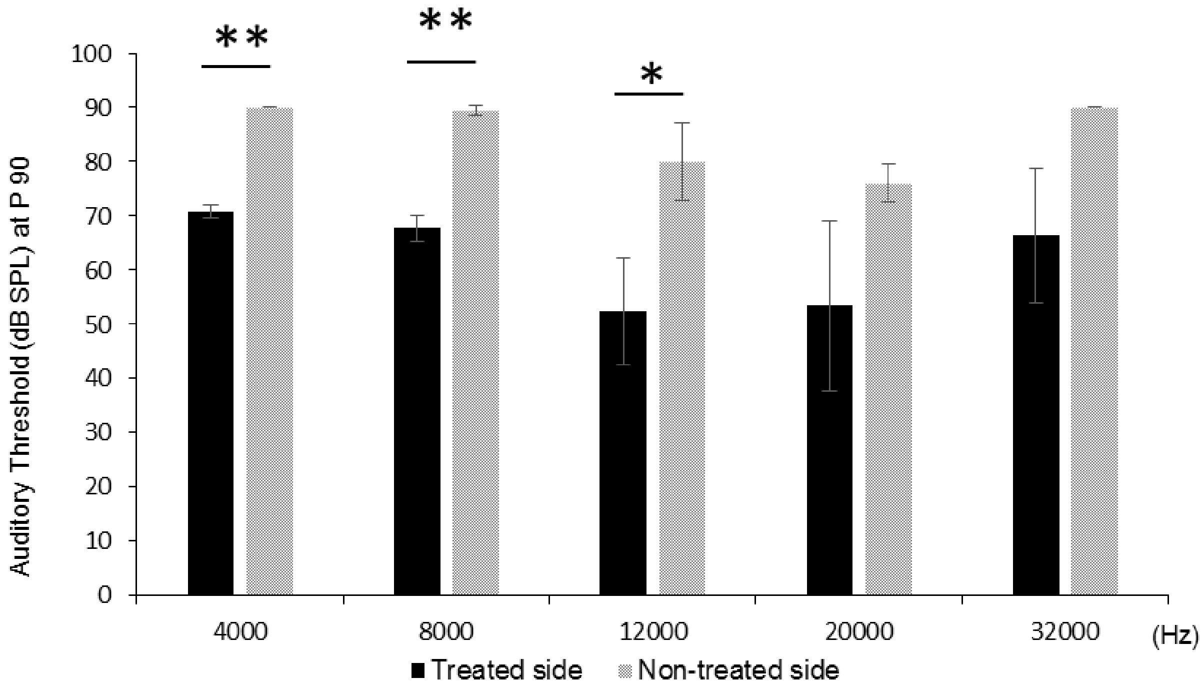

Supplemental Figure 5.

ABR testing results of *Slc26a4*$^{-/-}$ mice at P 90 after treatment. Statistically significant difference is found between the treated and the untreated sides in *Slc26a4*$^{-/-}$ mice at 4, 8 and 12 kHz. Each *n*=3. *: $P < 0.05$ (Student-t test)
